# Correction: Comparative dissection of the peripheral olfactory system of the Chagas disease vectors *Rhodnius prolixus* and *Rhodnius brethesi*

**DOI:** 10.1371/journal.pntd.0010294

**Published:** 2022-03-16

**Authors:** Florencia Campetella, Rickard Ignell, Rolf Beutel, Bill S. Hansson, Silke Sachse

The species name *Rhodnius brethesi* appears incorrectly throughout the article due to misidentification during the breeding process. The correct species name verified by genotyping is *Rhodnius neglectus*, which lives also in sylvatic areas (such as the Cerrado (savanna) region in Central Brazil) and nests on several species of palm trees. Both species share many similarities.
