# Supplementary figures and images for: Comparative dissection of the peripheral olfactory system of the Chagas disease vectors Rhodnius prolixus and Rhodnius brethesi
Source: PLoS Negl Trop Dis. 2021 Apr 15;15(4):e0009098. doi: 10.1371/journal.pntd.0009098 (PMC8078792; doi:10.1371/journal.pntd.0009098)

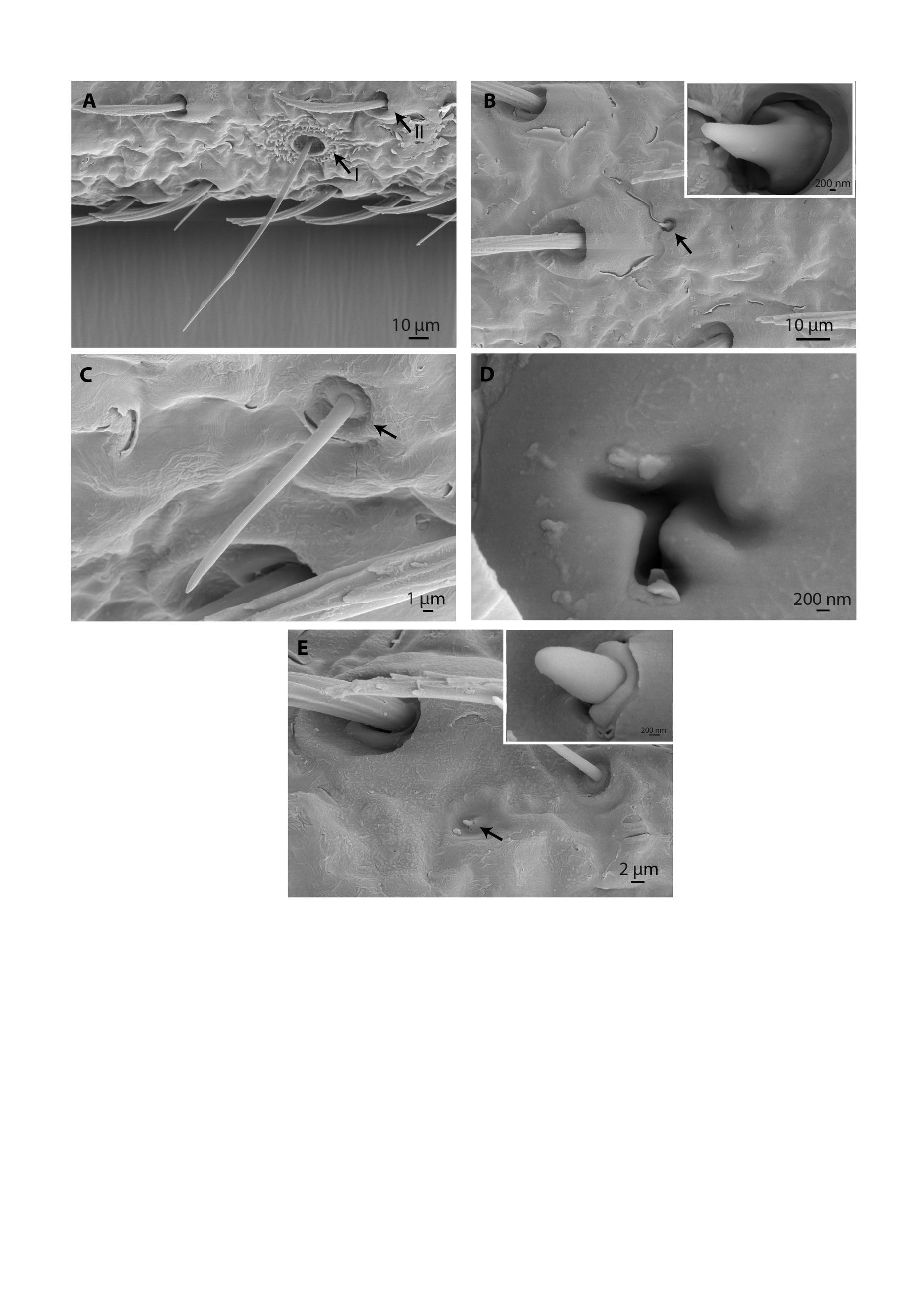

Supplement: S1 Fig — Arrows indicate (A) sensillum trichobothrium (I) and bristle II (II), (B) peg-in-pit sensilla, (C) bristle III, (D) ornamented pore, and (E) type 3 coeloconic sensilla, on the pedicel of the antenna. (TIF) [file pntd.0009098.s001.tif]

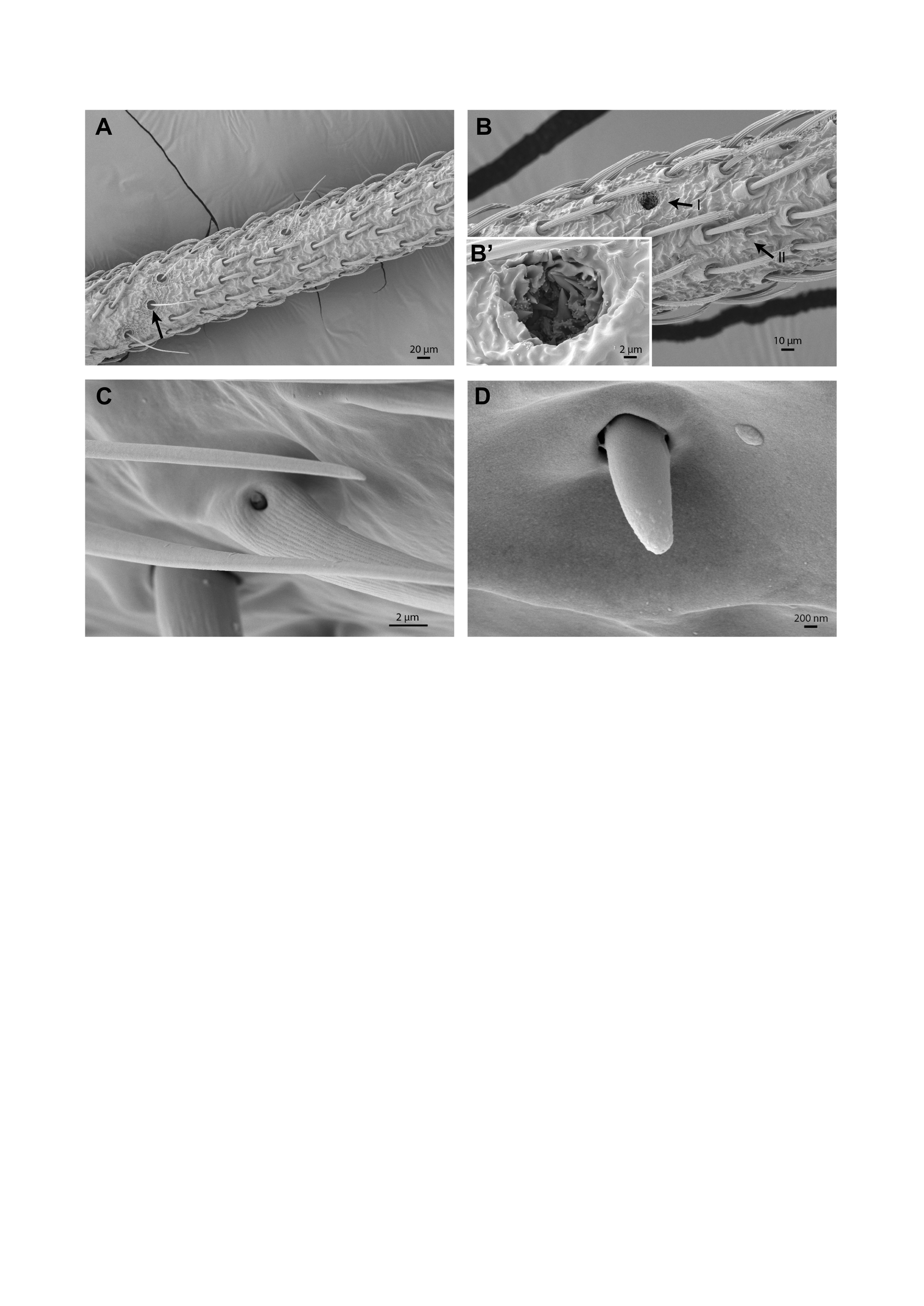

Supplement: S2 Fig — Arrows indicate (A) sensillum trichobothrium, (B) cave organ at the pedicel (I) and bristle III (II), (B’) detail of the cave organ, (C) basiconic (also known as thin-walled trichoid) sensillum presenting the ecdysis channel, (D) coeloconic sensillum. (TIF) [file pntd.0009098.s002.tif]

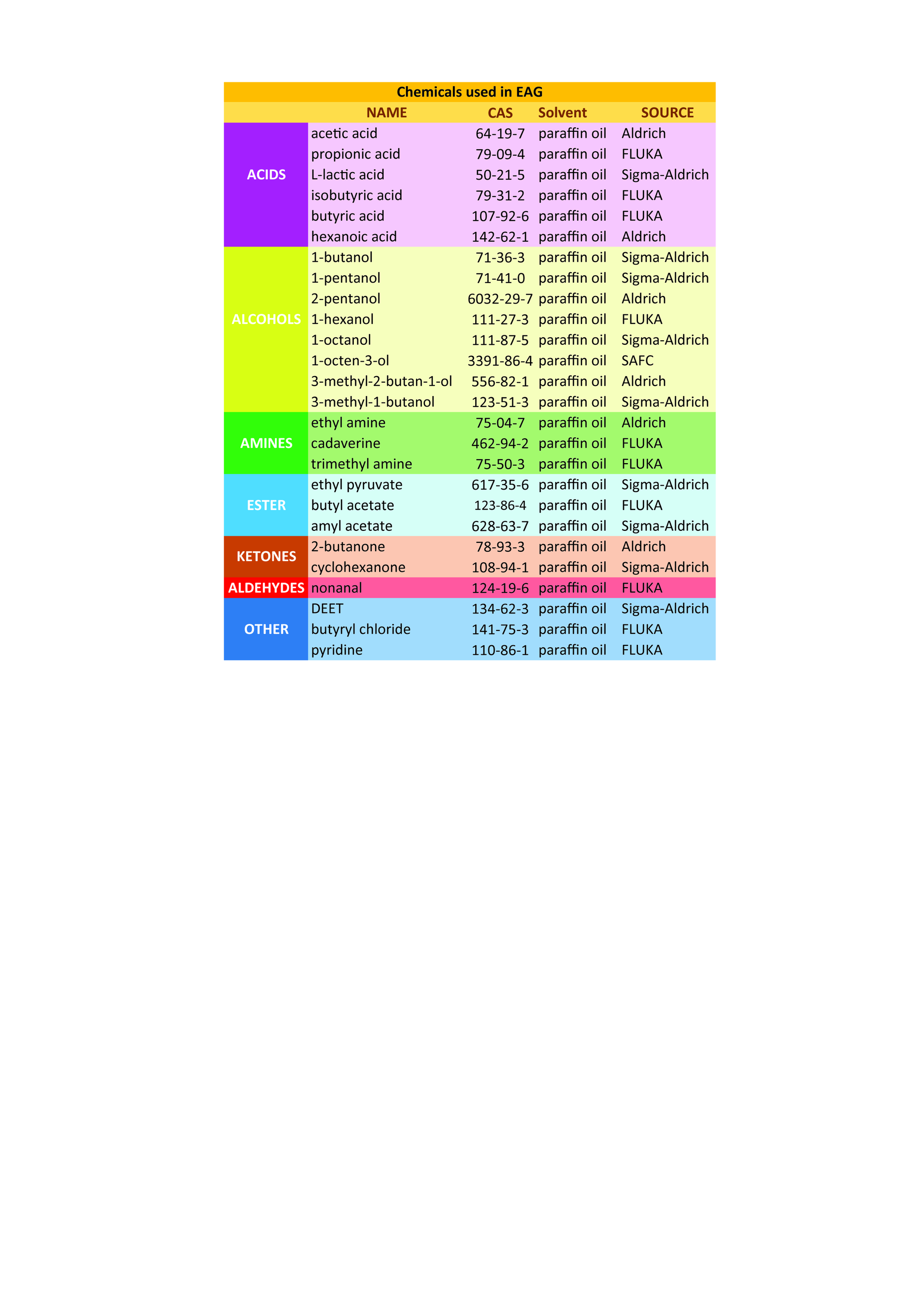

Supplement: S2 Table — (TIF) [file pntd.0009098.s004.tif]

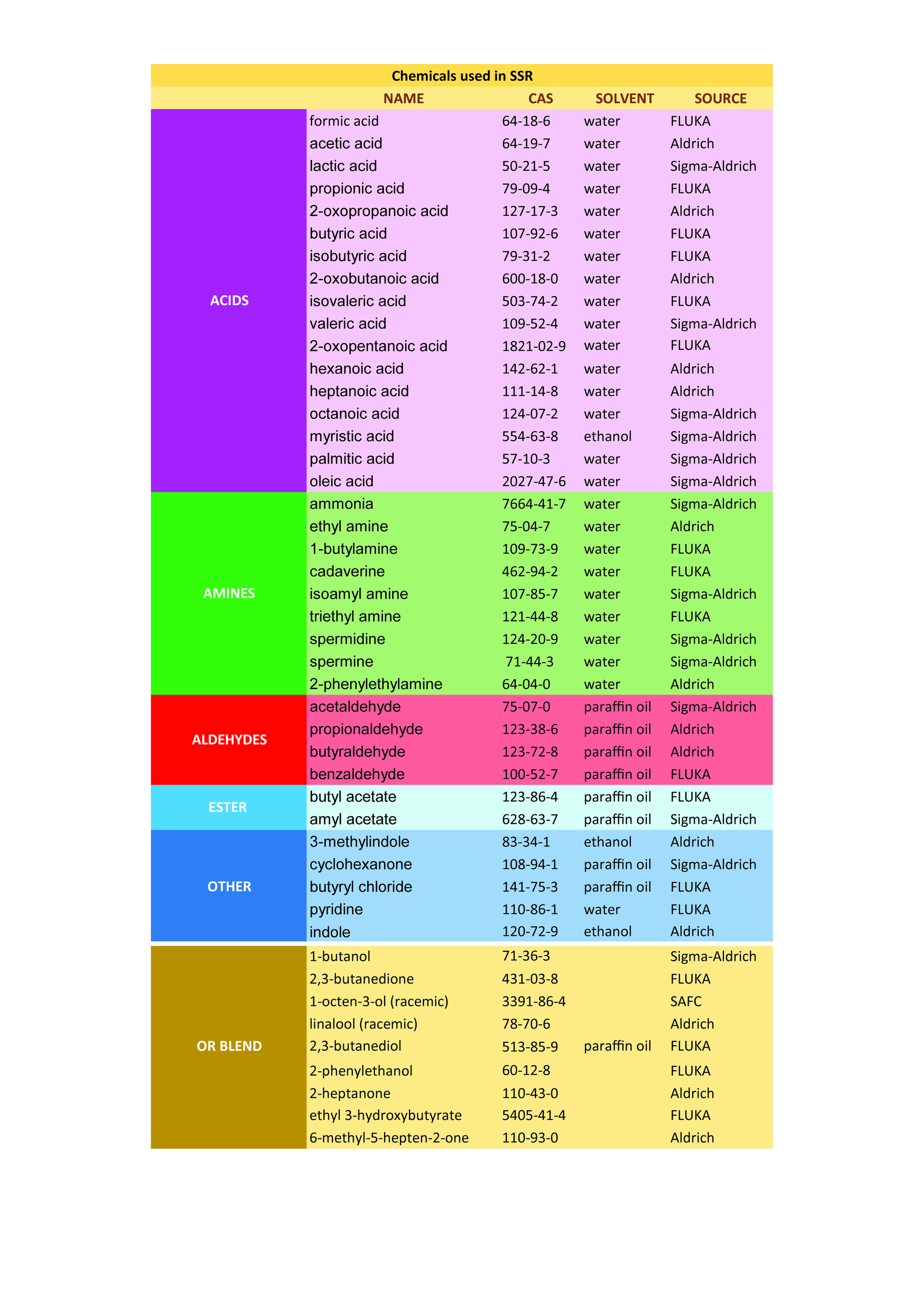

Supplement: S3 Table — (TIF) [file pntd.0009098.s005.tif]

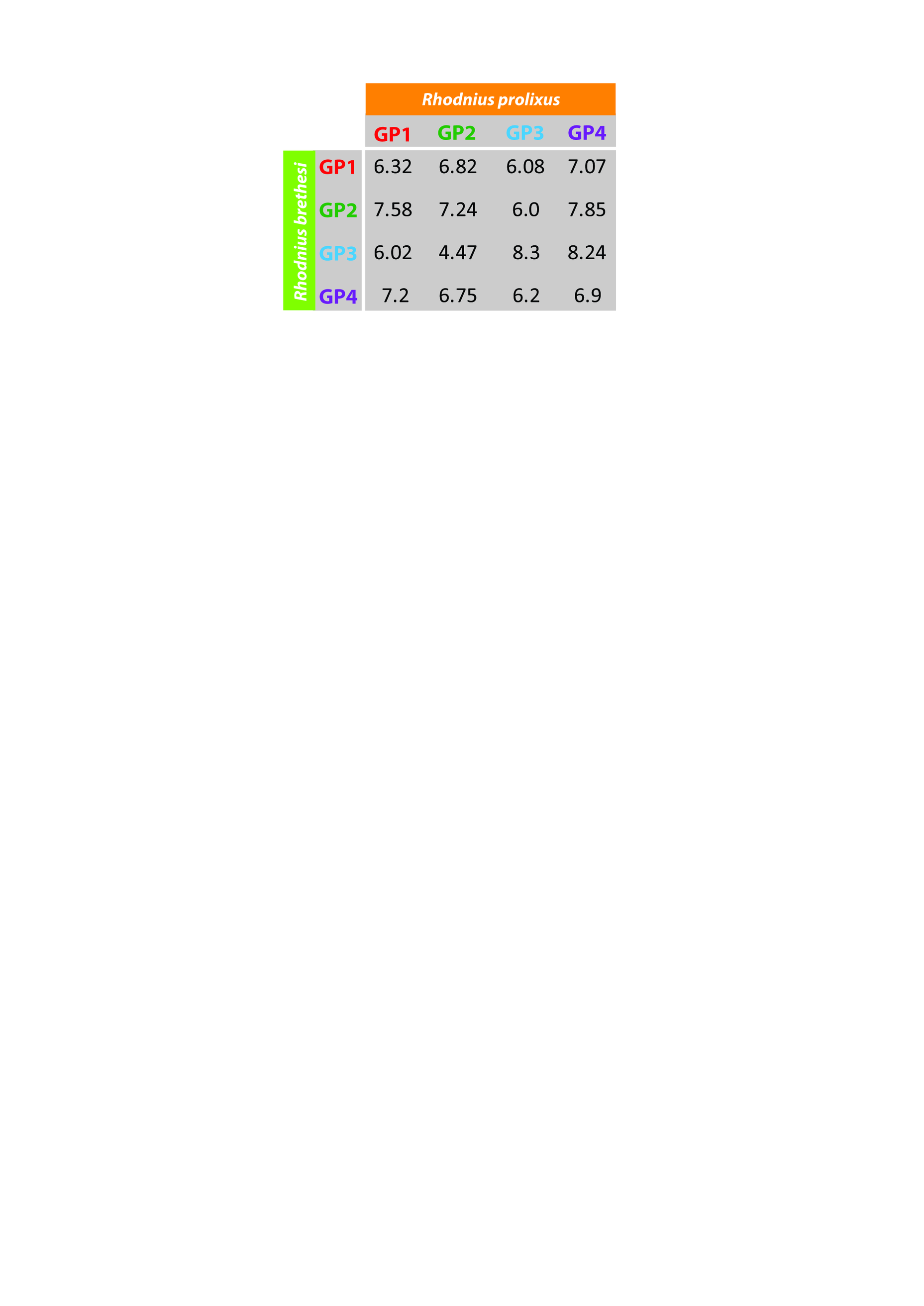

Supplement: S4 Table — (TIF) [file pntd.0009098.s006.tif]
