# Supplementary material for: Comparative dissection of the peripheral olfactory system of the Chagas disease vectors Rhodnius prolixus and Rhodnius brethesi
Source: PLoS Negl Trop Dis. 2021 Apr 15;15(4):e0009098. doi: 10.1371/journal.pntd.0009098 (PMC8078792; doi:10.1371/journal.pntd.0009098)
Supplement: Attachment — Submitted filename: Reviewer_Comments_Campetella_Rhodnius_final.pdf [file pntd.0009098.s007.pdf]

Response to reviewer comments

Campetella et al. (PNTD-D-02175)

Reviewer #1

Methods:

(1) The objectives of the study are clearly stated and the study design is appropriate. Sample sizes are generally adequated. However, concerning Fig 2B a non-parametric test may be more adequate given that n=3.

(1) Response: Thank you for your general positive feedback and your highly useful comments! A parametric unpaired t test was chosen after assessing the normal distribution of the data with a Shapiro-Wilk test, which allows analysis of small sample sizes.

(2) In Methods section basiconic sensilla are mentioned. However, later in the MS they seem to be called thin-walled trichoid. The nomenclature used should be clarified, citing previous papers and indicating the different names previously given to the same sensillum type (this is to allow the reader compare the information from this and other papers on the same sensillum type). I would suggest using the nomenclature by Shanbag, et al (1999) International Journal of Insect Morphology and Embryology 28 (1999) 377-397, which is in general agreement with Keil (1999) Insect Olfaction (Ed BS Hansson) and Altner, H., Prillinger, L., 1980. International Review of Cytology 67, 69-139, and used in triatomines in, for example, Ref 27 of the MS.

(2) Response: We apologize for being not consistent regarding the nomenclature of the different sensilla types studied in our MS. As suggested by the reviewer, we adopted the nomenclature by Shanbhag et al (1999), and referred to it in the text (lines 263ff, 279ff, 291ff) and corresponding figure (Figure 2).

(3) Pls check the formula around line 195, especially in line 201.

(3) Response: Thank you for the remark, the formula was corrected.

Results:

(4) In Fig 3 I did not understand which responses were significantly different from the negative control. Responses to CO_2 have been found but nothing is said about those responses. The resolution of figures 4, 5 should be improved. They are difficult to read.

(4) Response: In Figure 3, the filled bars represent significant responses compared to solvent control. This is stated in the Figure legend and now also added to the Results section (line 305). We apologize for not commenting on the response to CO_2 in the previous version of our MS. We now refer to the CO_2 responses in the Results and Discussion sections, as well as in the Methods section where we describe how the

delivery of CO_2 was implemented. Moreover, we have thoroughly changed the arrangements of Figs. 4-6 and added Fig. 7 in order to improve the text flow and to make the figures easier to read and follow.

Editorial suggestions/ minor modifications:

(5) In line 264 the following reference should be added: May-Concha, et al 2016. Infec. Genet. Evol. 40, 73–79

(5) Response: Thank you for providing the reference, which is indeed very relevant. It has now been added.

(6) In line 100 (or 106 or 104) the following reference should be added: Guidobaldi & Guerenstein 2016 Journal of medical entomology 53, 770-775

(6) Response: We apologize for missing this very important reference. It has now been added.

(7) There is a mistake in line 302. Aldehydes have also been tested (see your Ref 27).

(7) Response: The text has been amended accordingly.

(8) Check line 122 , it seems to contradict a previous sentence.

Line 59 Replace "its" for "a"

Correct TrYpanosoma in Author summary

(8) Response: The author summary has been revised and corrected.

Summary and General Comments:

(9) This MS deals with a comparison of the morphology and physiology of the olfactory system of two triatomine species, one considered domestic, the other sylvatic. A higher density of two types of olfactory sensilla in the sylvatic species was found compared to the domestic one. Also, the sylvatic species presented overall higher and broader olfactory responses than its domestic relative. (It is suggested that domestic species present a decreased olfactory function, in relation to the limited relevance of this sensorial input in their particular environment with limited olfactory cues.) In addition, knowledge about the olfactory responses on the antenna of triatomines has been extended, and this includes responses to "new" compounds and a better understanding of functional subtypes.

The morphology work is adequate and the experiments are correctly carried out. It would be interesting to continue this work, for example, by studying the dose-response characteristic of the ORNs studied. Also, an antennal response to CO_2 is reported for the first time in triatomines. It would be interesting to study this further.

In conclusion, this MS contributes new information on triatomines and in general add up to our knowledge on the olfactory system of hematophagous insects.

(9) Response: Thank you for your positive feedback! We agree with the reviewer that it would be interesting in future experiments to determine dose-response curves for a selected group of odorants, which would allow to characterize the responses of these ORNs further. We also agree that the antennal response to CO_2 is an interesting observation, as it still remains unknown which receptor mediates its detection. In preparatory experiments, application of CO_2 to the grooved peg sensilla did not result in a response. However, these suggested experiments are very time-consuming and should therefore be rather part of further investigations. We added these ideas regarding future studies to the discussion section of our manuscript (lines 536-538).

Reviewer #2

Methods:

(1) The objectives are clearly articulated and the study design is appropiate to address the stated objectives.

(1) Response: Thank you for the positive feedback and your highly constructive comments to our study, which we all incorporated into the revised version of our manuscript.

Results:

(2) The reading in this section was difficult. Probably because the Figures are quite messed up in the text, which makes reading tough. Therefore, I suggest (as a possibility) to group data differently. For example, data of figure 4A and those of figures 6C and D could be on the same template, where the averaged responses are shown (without function subtype discrimination). And, graphs of Figure 4B could be included along with data of Figure 6A-B. If so, this would need rewriting the text.

(2) Response: We thank the reviewer for suggesting to present and group the data in a different way. We have thoroughly revised the manuscript by changing the order and arrangements of the different figures and modifying the different sections, especially the results section, to improve text flow, comprehension and readability of the MS. We changed the arrangement and order of Figs. 4-6 and added Fig. 7.

Conclusions:

The authors could certainly further develop the limitations of the study and give alternative hypotheses to those provided (which I detail below).

(3) Response: We have thoroughly revised our hypotheses according to the reviewer's suggestions. Below we explain and respond in detail to each comment of the reviewer.

Summary and General Comments:

(4) In the manuscript "Comparative dissection of the peripheral olfactory system of the Chagas disease vectors *Rhodnius prolixus* and *Rhodnius brethesi*", the authors analyze morphological and physiological differences in the olfactory antennal sensilla between *R. prolixus* and *R. brethesi*. They found differences across species in the number of certain morphological types of olfactory sensilla (i.e. thin- and thick- walled trichoid sensilla) and also differences in the olfactory response profile of grooved peg (GP) sensilla. Whereas GP sensilla were found in similar numbers in both species, they showed different olfactory tuning responses. The results here provided are solid, novel and are a useful contribution to the sensory physiology of triatomine insects.

(4) Response: Thank you for your positive feedback!

(5) However, I have major concerns about the construction of the hypothesis and the hypothesis of this work. First, the authors claim that *R. prolixus* is a domiciliated species, which is not accurate. Whereas *R. brethesi* until now was only found in sylvatic environments, *R. prolixus* depending on the region can be found in sylvatic (living in palm trees, e.g. Colombia, Ecuador, Brazil, etc.) or in domestic environments (living in human dwellings, e.g. Guatemala). Therefore, *R. prolixus* does not represent the best example of an exclusively domiciliated species. An interesting comparison would be to analyze the antennal morphology and physiology of domiciliated and sylvatic *R. prolixus*.

(5) Response: Indeed, *R. prolixus* presents a broad distribution in Colombia, Ecuador, Nicaragua, however it presents a higher degree of domiciliation than other species of the genus Rhodnius (as evidenced by the number of houses infested by the insects), especially compared to *R. brethesi*. However, we agree that the differences we observe cannot solely and clearly be attributed to a possible domiciliation, as these species are also found in sylvatic areas and, as the reviewer points out, sylvatic species might also live in a restricted environment, like *R. brethesi*. To avoid over interpretation of our results, we restricted the hypothesis to a comparison between two species of triatomines, one being sylvatic and the other having a broader distribution in sylvatic, domestic and peridomestic areas. Furthermore, we agree that a comparison between domestic and sylvatic triatomines would be interesting to assess intraspecific differences in sensory perception, and would certainly increase our knowledge about the role the environment has in shaping olfactory detection in triatomines. Our study, however, was aimed at examining inter-specific differences between triatomines, which might result from speciation. We decided to focus on *R. prolixus* as it is broadly distributed and is one of the triatomine species that received most attention in the past. Based on previous work, we expanded, not without limitation, the knowledge we have about this species. Our choice of *R. brethesi* was based on the fact that this species has been found only in sylvatic environments. We asked ourselves: how different are the responses from this sylvatic species, compared to those ones of *R. prolixus*? We hope that the modifications of the text and our hypotheses now capture our research question more precisely.

(6) Second, the authors postulate that sylvatic species (*R. brethesis*) have higher number of sensilla and a larger olfactory tuning of sensory neurons than domicialiated species (*R. prolixus*) due to their need for an active search for hosts, a requirement that would be higher in sylvatic species than in domiciliated ones. This statement, on which the general hypothesis of the work is based, is weak. In fact, we know that sylvatic species also live in close association with their hosts, probably spending their whole lifecycle in the same vertebrate nest. In these nests they form large colonies and several cohorts coexist. Therefore, asserting that sylvatic triatomines explore and search more actively than domiciliated species, and consequently their need for a more complex sensory machinery than domiciliated ones, is not a solid argument or hypothesis for triatomine species as it might be for other insect species. The differences

found with respect to the sensory tools could simply be attributed to species-specific differences related to different host preferences.

(6) Response: We agree that the differences found could also be attributed to species-specific differences in hosts. We hope our thorough revision of the text now reflects this hypothesis. Still, it remains as an open question whether sylvatic insects need to discriminate specific olfactory cues against a background containing a higher amount of volatiles, even if they live in close association with a host.

(7) Lastly, reading was difficult. Probably because the Figures are quite messed up in the text, which makes reading tough. Therefore, I suggest (as a possibility) to group the data differently. For example, data of Figure 4A and those of Figures 6C and D could be on the same template, where the averaged responses are shown (without function subtype discrimination). And, graphs of Figure 4B could be included along with data of Figure 6A-B. If so, this would need rewriting the text.

(7) Response: We are sorry for the difficulties caused, and thank the reviewer for the constructive suggestions. The figures and text have been thoroughly revised. We decided to re-arrange the figures in a different manner. What used to be Figure 6C, D is now Figure 4, Figure 5A is now Figure 5, Figure 4A is now under Figure 6 and we combined Figures 4B,C, Figure 6A, B and Figure 5B into a new figure (i.e. Figure 7). We hope that this new arrangement improves the legibility of the manuscript. The text has been accordingly rewritten.

(8) lines 47-48 "American Tripanosomiasis, also known as Chagas disease, is a disease which no one speaks out…." This affirmation sounds weird when the WHO produces annual reports informing to the scientific and non-scientific community about the statistics of the disease (% of people affected, transmission rates, preventive actions and policies, etc..). Chagas disease is probably less known in Europe or Asia, but it is not the case in the Americas. Moreover, the Center for Disease Control and Prevention (CDC) of the United States considered the Chagas disease as 1 of 5 parasitic infections to be targeted as priority for public health (CDC, 2018).

(8) Response: We have revised the author summary and avoided to mention that Chagas disease represents a hardly known disease, as it has been well characterized and documented.

(9) line 52 "… and to get their vital blood meal, while infecting them at the same time" this is not precise but is confusing and can be misunderstood. If kissing bugs do not defecate during blood intake, there are no chances of infecting the host, given the parasites are only present in feces and not in the salivary glands. Thus, they can take a small blood meal and leave the host without defecating. I suggest editing the text, to avoid confusion and misunderstandings.

(9) Response: We are sorry for the phrasing and confusion. The author summary has been corrected.

(10) line 66 replace for Chagas disease

(10) Response: Thank you for the remark; it has been amended. We also decided to uniformly use “Chagas disease” throughout the text.

(11) lines 106-107 "…largely unknown" ? SSR have been carried out before this work, in T. infestans (Guerenstein & Guerin 2001 Diehl et al 2003, Taneja & Guerin 1997, Mayer 1968, Bernard 1974, Guerenstein 1999), R. prolixus (Reisenman 2014) Several and previous studies to this work have pointed out the role of the antenna and the olfactory in odour detection. In consequence this sentence is not precise. I suggest editing it.

(11) Response: We apologize for our phrasing that understated previous significant work in the field. The text has been revised accordingly and the missing references have been added (lines 104-108). The intention was to point out that most of SSR studies were done in *T. infestans* and not in *R. prolixus*. As we discuss, there might be differences in responses between these two species.

(12) lines 109 -111 This is not precise either. Like domestic triatomines sylvatic species also live in close association with their hosts. Sylvatic species live in vertebrate nest, where they can spend in the same nest their entire lifecycle, forming large colonies. Therefore, I disagree with the authors in claiming that sylvatic triatomines explore and search for hosts more actively than domiciliated species.

(12) Response: The comment is accurate and we agree with the reviewer that sylvatic species can also live in restricted habitats and present very narrow host choices. Such is indeed the case for *R. brethesi*. We have revised this part to avoid confusions (lines 109ff).

(13) line 278. You stated that you've identified 2 new types of sensilla: 1- a peg-in-pit sensillum and a type of coeloconic sensillum. Please specify whether it was an eventual finding or not. If not, please specify in how many preparations you found them.

(13) Response: The finding of new sensilla types was indeed an eventual finding, which is now specified in the text as suggested.

(14) lines 294-295: In figure 2, which is the point of the micrographs shown above the bars of density quantification of sensilla? Please refer to them or exclude them.

(14) Response: The micrographs show the confocal scans that were used to quantify the sensillum density. However, following the reviewer’s suggestion, we decided to exclude them, as they did not significantly contribute to the understanding of the sensilla quantification.

(15) It is shown in Fig.3 that you've tested the response to CO2, please detail in Mat & Met how did you offer the CO2 to the insects, and which was the concentration offered?

(15) Response: We apologize for not explaining how CO2 was exactly applied and what concentration was used in our study. We have now added these details to the Materials and Method and Results sections (lines 157-158, 170-172, 313-315).

(16) lines 344-346 : You stated "While only 37% of the odor-sensillum combinations in *R. prolixus* yielded responses >15 spikes s-1 above solvent response, all combinations did in *R. brethesi* (Fig. 4, Fig. 5). " However this is not easily to visualize, on the contrary, it looks the opposite. To facilitate cross-species comparisons, I strongly suggest, in Figure 5, putting the Y axes on the same scale.

(16) Response: As the absolute number of odor-sensillum combinations vary between species, we have revised Figure 5 using the same scaling for both species, as suggested by the reviewer, and the corresponding total percentages (i.e. cumulative sum) have been corrected.

(17) line 283. Instead of FigS2E wouldn't it be replace for FigS1E?

(17) Response: Yes, this has been corrected.

(18) lines 492 - 494 You stated "However, it is conceivable that these chemicals are detected by organs other than the antenna, as the odorant co-receptor orco is expressed also in tarsi, genitalia and rosti". No olfactory sensilla have been identified in tarsi, genitalia or rostrum of triatomine bugs. The presence of odorant-related receptors in these appendages may not necessarily be related to an olfactory function. Please rephrase.

(18) Response: We agree with the reviewer’s comment and have rephrased this sentence (lines 483-486). However, we would like to point out that in *Rhodnius prolixus* not only RproOrco but also RproOR13, RproOR20 and RproOR104 were found to be expressed in rostri, tarsi, tibial pads and ovipositor or genitalia of females and males, respectively (Latorre-Estivalis et al., Insect Biochem. Molec., 2016). This finding highly suggests an olfactory function – though perhaps limited – in these tissues. Morphological studies have shown the presence of sensilla in some of these tissues (Catala, J., Morphology, 1996; Silva et al., Mem Inst Oswaldo Cruz., 2003; Santos-Mallet et al., Mem Inst Oswaldo Cruz., 2005; da Rosa et al., Mem Inst Oswaldo Cruz., 2010), but their olfactory function was actually not probed. In other heteropteran species, sensilla with olfactory function have been identified in the rostrum, genitalia and tarsi (e.g. Brozek and Zettel, European J. Entomology, 2014) and widespread expression of chemosensory receptors has been reported for other insect species (Xia and Zwiebel, Insect Biochem. Molec, 2006; Olivier et al., Insect Mol. Biol., 2011; Hull et

al., Arch. Insect Biochem., 2012; Wang et al., Parasitol. Res., 2012; Yang et al., Int. J. Biol. Sci., 2012).

(19) lines 557- 559 You stated "It seems conceivable that the sylvatic *R. brethesi* uses olfaction to discriminate between a higher number of hosts and nest sites. In contrast, the domestic *R. prolixus* might encounter a rather limited number of olfactory stimuli." I would like to remark that *R. brethesi* infests only one type of palm tree (Leopoldinia piassaba). Consequently, this may also suggest that this wild triatomine has special characteristics in its sensory system allowing specificity for this ecotope.

(19) Response: We thank the reviewer for mentioning this possibility, which we added now to the discussion section (lines 549ff). In general, due to our lack of knowledge on the chemical ecology of sylvatic species, and of *R. brethesi* in particular, whether our findings reflect an adaptation to their ecotope or not is difficult to say.

(20) lines 571- 572 You stated "… the condition found in the sylvatic species represents the ancestral character state in the subfamily, whereas a derived reduced condition is linked with a more or less close association with humans." It would be interesting to compare morphological and physiological differences between domiciliated and sylvatic *R. prolixus*.

(20) Response: We agree with the reviewer that such a comparison would be indeed interesting and insightful. However, identifying truly wild *R. prolixus* insects might be challenging (especially during the current pandemic situation), as it seems insects of this species present a high mobility between ecotopes (Abad-Franch et al., An Acad Bras Cienc., 2005; Guhl et al., Mem Inst Oswaldo Cruz., 2009; Poveda et al., Infect Genet Evol., 2017). Moreover, such a comparison would be beyond the scope of our current study. However, it remains as a priority in future studies and is now mentioned in our discussion (lines 553ff).
